# Computational Methods for Parameter Identification in 2D Fractional System with Riemann–Liouville Derivative

**DOI:** 10.3390/s22093153

**Published:** 2022-04-20

**Authors:** Rafał Brociek, Agata Wajda, Grazia Lo Sciuto, Damian Słota, Giacomo Capizzi

**Affiliations:** 1 Department of Mathematics Applications and Methods for Artificial Intelligence, Faculty of Applied Mathematics, Silesian University of Technology, 44-100 Gliwice, Poland; damian.slota@polsl.pl; 2 Institute for Chemical Processing of Coal, 41-803 Zabrze, Poland; awajda@ichpw.pl; 3 Department of Mechatronics, Silesian University of Technology, Akademicka 10a, 44-100 Gliwice, Poland; grazia.losciuto@polsl.pl or; 4 Department of Electrical, Electronics and Informatics Engineering, University of Catania, Viale Andrea Doria, 6, 95125 Catania, Italy; gcapizzi@dees.unict.it

**Keywords:** inverse problem, fractional system, fractional derivative, parameter identification, fractional differential equation, heuristic algorithm, computational methods

## Abstract

In recent times, many different types of systems have been based on fractional derivatives. Thanks to this type of derivatives, it is possible to model certain phenomena in a more precise and desirable way. This article presents a system consisting of a two-dimensional fractional differential equation with the Riemann–Liouville derivative with a numerical algorithm for its solution. The presented algorithm uses the alternating direction implicit method (ADIM). Further, the algorithm for solving the inverse problem consisting of the determination of unknown parameters of the model is also described. For this purpose, the objective function was minimized using the ant algorithm and the Hooke–Jeeves method. Inverse problems with fractional derivatives are important in many engineering applications, such as modeling the phenomenon of anomalous diffusion, designing electrical circuits with a supercapacitor, and application of fractional-order control theory. This paper presents a numerical example illustrating the effectiveness and accuracy of the described methods. The introduction of the example made possible a comparison of the methods of searching for the minimum of the objective function. The presented algorithms can be used as a tool for parameter training in artificial neural networks.

## 1. Introduction

Fractional calculus is widely used in various fields of science and technology, e.g., in the design of sensors, in signal processing, and network sensors [1,2,3,4,5]. In the paper [2], authors describe the use of fractional calculus for artificial neural networks. Fractional derivatives are mainly used for parameter training using optimization algorithms, system synchronization, and system stabilization. As the authors quote, such systems have been used in unmanned aerial vehicles (UAVs), circuit realization robotics, and many other engineering applications. The paper [3] covers applications of fractional calculus in sensing and filtering domains. The authors present the most important achievements in the fields of fractional-order sensors, fractional-order analogs, and digital filters. In [5], they present a new fractional sensor based on a classical accelerometer and the concepts of fractional calculus. In order to achieve this, two synthesis methods were presented: the successive stages follow an identical analytical recursive formulation, and in the second method, a PSO algorithm determines the fractional system elements numerically.

In addition to applications in electronics, neural networks, and sensors, fractional calculus is also used in modeling of thermal processes [6,7], in modeling of anomalous diffusion [8,9], in medicine [10], and also in control theory [11,12]. Authors of the study in [6] model heat transfer in a two-dimensional plate using Caputo operator. Theoretical results are verified by experimental data from a thermal camera. It is shown that the fractional model is more accurate than the integer-order model in the sense of mean square error cost function.

Often in applications of fractional calculus, differential equations with fractional derivatives have to be solved numerically. This is the reason for the importance of developing algorithms for solving this type of problem. A lot of papers presenting numerical solutions of fractional partial differential equations have been published in recent years. In the paper [13], the author used the artificial neural network in the construction of a solution method for the one-phase Stefan problem. In turn, Ref. [14] presented an algorithm for the solution of fractional-order delay differential equations. Bu et al., in [15], presented a space–time finite element method to solve a two-dimensional diffusion equation. The paper describes a fully discrete scheme for the considered equation. Authors also presented a theorem regarding existence, stability of the presented method, and error estimation with numerical examples. Another interesting study is [16], in which the ADI method to solve fractional reaction–diffusion equations with Dirichlet boundary conditions was described. The authors used a new fractional version of the alternating direction implicit method. A numerical example was also presented.

In the paper, authors present a solution to the inverse problem consisting of the appropriate selection of the model input parameters in such a way that the system response adjusts to the measurement data. Inverse problems are a very important part of all sorts of engineering problems [17]. In [18], the inverse problem is considered for fractional partial differential equation with a nonlocal condition on the integral type. The considered equation is a generalization of the Barenblatt–Zheltov–Kochina differential equation, which simulates the filtration of a viscoelastic fluid in fractured porous media. In [19], the authors considered two inverse problems with a fractional derivative. The first problem is to reconstruct the state function based on the knowledge of its value and the value of its derivative in the final moments of time. The second problem consists of recreating the source function in fractional diffusion and wave equations. Additional information are the measurements in a neighborhood of final time. The authors prove the uniqueness of the solution to these problems. Finally, the authors derive the explicit solution for some particular cases. In the paper [20], the fractional heat conduction inverse problem is considered, consisting of finding heat conductivity in presented model. The authors also compare two optimization methods: iteration method and swarm algorithm.

The learning algorithm constitutes the main part of deep learning. The number of layers differentiates the deep neural network from shallow ones. The higher the number of layers, the deeper it becomes. Each layer can be specialized to detect a specific aspect or feature. The goal of the learning algorithm is to find the optimal values for the weight vectors to solve a class of problem in a domain. Training algorithms aim to achieve the end goal by reducing the cost function. While weights are learned by training on the dataset, there are additional crucial parameters, referred to as hyperparameters, that are not directly learned from the training dataset. These hyperparameters can take a range of values and add complexity of finding the optimal architecturenand model [21]. Deep learning can be optimized in different areas. The training algorithms can be fine-tuned at different levels by incorporating heuristics, e.g., for hyperparameter optimization. The time to train a deep learning network model is a major factor to gauge the performance of an algorithm or network, so the problem of the training optimization in a deep learning application can be seen as the solution of an inverse problem. In fact, the inverse problem consists of selecting the appropriate model input parameters in order to obtain the desired data on the output. To solve the problem, we create an objective function that compares the desired values (target) with the network outputs calculated for the determined values of the searched parameters (weights). Finding the minimum of the objective function, we find the sought weights.

In this paper, in Section 2, a system consisting of a 2D fractional partial differential diffusion equation with Riemann–Liouville derivative is presented. Dirichlet boundary conditions were added to the equation. This type of model can be used for the designing process of heat conduction in porous media. In Section 2.2, a numerical scheme of the considered equation is presented based on the alternating direction implicit method (ADIM). In Section 3, the inverse problem is formulated. It consists of identification of two parameters of the presented model based on measurements of state function in selected points of the domain. The inverse problem has been reduced to solving the optimization problem. For this purpose, two algorithms were used and compared: probabilistic ant colony optimization (ACO) algorithm and deterministic Hooke–Jeeves (HJ) method. Section 4 presents a numerical example illustrating the operation of the described methods. Section 5 provides the conclusions.

## 2. Fractional Model

This section consists of a description of the considered anomalous diffusion model which is considered with a fractional derivative, and then we present a numerical algorithm solving the presented differential equation.

### 2.1. Model Description

Models using fractional derivatives have recently been widely used in various engineering problems, e.g., in electronics for modeling a supercapacitor, in mechanics for modeling heat flow in porous materials, in automation for describing problems in control theory, or in biology for modeling drug transport. In this study, we consider the following model of anomalous diffusion:

(1) $$\begin{array}{c} c\varrho \frac{\partial u(x,y,t)}{\partial t}=\frac{\partial }{\partial x}\left(\lambda \left(x,y\right)\frac{\partial^{\alpha }u\left(x,y,t\right)}{\partial x^{\alpha }}-\lambda \left(x,y\right)\frac{\partial^{\alpha }u\left(x,y,t\right)}{\partial {\left(-x\right)}^{\alpha }}\right)\\ +\frac{\partial }{\partial y}\left(\lambda \left(x,y\right)\frac{\partial^{\beta }u\left(x,y,t\right)}{\partial y^{\beta }}-\lambda \left(x,y\right)\frac{\partial^{\beta }u\left(x,y,t\right)}{\partial {\left(-y\right)}^{\beta }}\right)+f\left(x,y,t\right), \end{array}$$

(2) $$\begin{array}{c} {u\left(x,y,t\right)|}_{\partial \Omega }=0,\;\;t\in \left(0,T\right],\\ {u\left(x,y,t\right)|}_{t=0}=\varphi \left(x,y\right),\;\;\left(x,y\right)\in \Omega . \end{array}$$

The differential Equation (1) describes the anomalous diffusion phenomenon (e.g., heat conduction in porous materials [22,23,24]), and is defined in the area Ω×T, where (x,y)∈Ω, c,ϱ,λ>0 are parameters defining material properties, *u* is a state function, and *f* is an additional component in the model. Using the terminology taken from the theory of heat conduction, we can write that *c* is the specific heat, ϱ is the density, λ is the heat conduction coefficient, and the function *f* describes the additional heat source. All parameters are multiplied by the constants by the value of one and the units that ensure the compatibility of the units of the entire equation. The state function *u* describes the temperature distribution in time and space. The Equation (2) define the initial boundary conditions necessary to uniquely solve the differential equation. It is assumed that at the boundary the *u* state function has the value 0, and at the initial moment the value of the *u* function is determined by the well-known φ function. In the Equation (1), there also occurs fractional derivative of α and β order. In the model under consideration, these derivatives are defined as Riemann–Liouville [25] derivatives:

(3) $$\frac{\partial^{\alpha }u\left(x,y,t\right)}{\partial x^{\alpha }}=\frac{1}{\Gamma (1-\alpha )}\frac{\partial }{\partial x}\int_{0}^{x}{\left(x-\xi \right)}^{-\alpha }u\left(\xi ,y,t\right)\;d\xi ,$$

(4) $$\frac{\partial^{\alpha }u\left(x,y,t\right)}{\partial {\left(-x\right)}^{\alpha }}=\frac{-1}{\Gamma (1-\alpha )}\frac{\partial }{\partial x}\int_{x}^{L_{x}}{\left(\xi -x\right)}^{-\alpha }u\left(\xi ,y,t\right)\;d\xi .$$

The Formula (3) defines the left derivative, and the Formula (4) defines the right derivative. In both cases, they assume that α∈(0,1). In addition, the derivative of *y* of β order in the Equation (1) is defined as the Riemann–Liouville derivative.

### 2.2. Numerical Solution of Direct Problem

Now, let us present the numerical solution of the model defined by Equations (1) and (2). If we have all the data about the model, such as parameters c,ϱ,λ,α,β, initial boundary conditions, and geometry of the area, by solving the Equation (1), we solve the direct problem. In order to solve the problem under consideration, we write the Equation (1) as follows:

(5) $$\begin{array}{c} c\varrho \frac{\partial u(x,y,t)}{\partial t}=\left(\lambda_{x1}\left(x,y\right)\frac{\partial^{\alpha +1}u\left(x,y,t\right)}{\partial x^{\alpha +1}}+\lambda_{x2}\left(x,y\right)\frac{\partial^{\alpha +1}u\left(x,y,t\right)}{\partial {\left(-x\right)}^{\alpha +1}}\right)\\ +\left(\frac{\partial \lambda_{x1}\left(x,y\right)}{\partial x}\frac{\partial^{\alpha }u\left(x,y,t\right)}{\partial x^{\alpha }}-\frac{\partial \lambda_{x2}\left(x,y\right)}{\partial x}\frac{\partial^{\alpha }u\left(x,y,t\right)}{\partial {\left(-x\right)}^{\alpha }}\right)\\ +\left(\lambda_{y1}\left(x,y\right)\frac{\partial^{\beta +1}u\left(x,y,t\right)}{\partial y^{\beta +1}}+\lambda_{y2}\left(x,y\right)\frac{\partial^{\beta +1}u\left(x,y,t\right)}{\partial {\left(-y\right)}^{\beta +1}}\right)\\ +\left(\frac{\partial \lambda_{y1}\left(x,y\right)}{\partial y}\frac{\partial^{\beta }u\left(x,y,t\right)}{\partial y^{\beta }}-\frac{\partial \lambda_{y2}\left(x,y\right)}{\partial y}\frac{\partial^{\beta }u\left(x,y,t\right)}{\partial {\left(-y\right)}^{\beta }}\right)\\ +f(x,y,t). \end{array}$$

Then, we discretize the area $\Omega \times \left[0,T\right]=\left[0,L_{x}\right]\times \left[0,L_{y}\right]\times \left[0,T\right]$ by creating an uniform mesh in each of the dimensions. Let us assume the following symbols: $\Delta t=\frac{T}{N}$, $t^{k}=k\Delta t$, k=0.1,…N, $\Delta x=\frac{L_{x}}{M_{x}}$, $x_{i}=i\Delta x$, $i=0.1,\ldots ,M_{x}$, $\Delta y=\frac{L_{y}}{M_{y}}$, $y_{j}=j\Delta y$, $j=0.1,\ldots ,M_{y}$, where $N,M_{x},M_{y}\in N$ are mesh sizes, and $\left(t_{k},x_{i},y_{j}\right)$ are points of mesh. The values of the u,f,λ functions in the grid points are labeled as $u_{i,j}^{k},f_{i,j}^{k},\lambda_{i,j}$. We approximate the Riemann–Liouville derivative using the shifted Grünwald formula [26]:

(6) $${\left.\frac{\partial^{\alpha }u\left(x,y,t\right)}{\partial x^{\alpha }}\right|}_{\left(x_{i},y_{j},t^{k}\right)}\approx \frac{1}{{\left(\Delta x\right)}^{\alpha }}\sum_{l=0}^{i+1}\omega_{l}^{\alpha }u\left(x_{i-l+1},y_{j},t^{k}\right),$$

(7) $${\left.\frac{\partial^{\alpha }u\left(x,y,t\right)}{\partial {\left(-x\right)}^{\alpha }}\right|}_{\left(x_{i},y_{j},t^{k}\right)}\approx \frac{1}{{\left(\Delta x\right)}^{\alpha }}\sum_{l=0}^{M_{x}-i+1}\omega_{l}^{\alpha }u\left(x_{i+l-1},y_{j},t^{k}\right),$$

where

$$\omega_{0}^{\alpha }=\frac{\alpha }{2}g_{0}^{\alpha },\;\;\omega_{l}^{\alpha }=\frac{\alpha }{2}g_{l}^{\alpha }+\frac{2-\alpha }{2}g_{l-1}^{\alpha },\;\;l=1,2,\ldots ,$$

$$g_{0}^{\alpha }=1,\;\;g_{l}^{\alpha }=\left(1-\frac{\alpha +1}{l}\right)g_{l-1}^{\alpha }\;\;l=1,2,\ldots$$

Similarly, we can approximate the fractional derivative to the spatial variable *y*. In the case of the derivative over time, we use the difference quotient:

(8) $${\left.\frac{\partial u(x,y,t)}{\partial t}\right|}_{\left(x_{i},y_{j},t^{k+\frac{1}{2}}\right)}\approx \frac{u\left(x_{i},y_{j},t^{k+1}\right)-u\left(x_{i},y_{j},t^{k}\right)}{\Delta t}.$$

Let us use the following notation:

(9) $$\delta_{x}^{\alpha }u_{i,j}^{k}=\frac{1}{2{\left(\Delta x\right)}^{\alpha }}\left[\lambda_{i,j}^{{}_{x}^{\prime }}\sum_{l=0}^{i+1}\omega_{l}^{\alpha }u_{i-l+1,j}^{k}-\lambda_{i,j}^{{}_{x}^{\prime }}\sum_{l=0}^{M_{x}-i+1}\omega_{l}^{\alpha }u_{i+l-1,j}^{k}\right]$$

(10) $${\bar{\delta }}_{x}^{\alpha +1}u_{i,j}^{k}=\frac{1}{2{\left(\Delta x\right)}^{\alpha +1}}\left[\lambda_{i,j}\sum_{l=0}^{i+1}\omega_{l}^{\alpha +1}u_{i-l+1,j}^{k}+\lambda_{i,j}\sum_{l=0}^{M_{x}-i+1}\omega_{l}^{\alpha +1}u_{i+l-1,j}^{k}\right],$$

where $\lambda_{i,j}^{{}_{x}^{\prime }}$ denotes the first-order derivative (at $\left(x_{i},y_{j}\right)$) over the λ function with respect to the *x* variable. We assume analogous symbols for the *y* variable. After using the Formulas (6)–(10) and some transformations, the difference scheme for the Equation (5) can be written in the following form:

(11) $$\begin{array}{c} \left(1-\frac{\Delta t}{c\varrho }{\bar{\delta }}_{x}^{\alpha +1}-\frac{\Delta t}{c\varrho }\delta_{x}^{\alpha }-\frac{\Delta t}{c\varrho }{\bar{\delta }}_{y}^{\beta +1}-\frac{\Delta t}{c\varrho }\delta_{y}^{\beta }\right)u_{i,j}^{k+1}\\ =\left(1+\frac{\Delta t}{c\varrho }{\bar{\delta }}_{x}^{\alpha +1}+\frac{\Delta t}{c\varrho }\delta_{x}^{\alpha }+\frac{\Delta t}{c\varrho }{\bar{\delta }}_{y}^{\beta +1}+\frac{\Delta t}{c\varrho }\delta_{y}^{\beta }\right)u_{i,j}^{k}+\frac{\Delta t}{c\varrho }f_{i,j}^{k+\frac{1}{2}}, \end{array}$$

where $i=1,2,\ldots ,M_{x}-1$, $j=1,2,\ldots ,M_{y}-1$ and k=0,1,…,N−1.

In order to simplify the description of the numerical algorithm to be implemented, we present the difference schema (11) in matrix form, so we introduce the following matrices:

$$R_{x}\left(l\right)={\left(r_{i,j}^{x}\left(l\right)\right)}_{\left(M_{x}-1\right)\times \left(M_{x}-1\right)},\;\;l=1,2,\ldots ,M_{y}-1,$$

$$R_{y}\left(l\right)={\left(r_{i,j}^{y}\left(l\right)\right)}_{\left(M_{y}-1\right)\times \left(M_{y}-1\right)},\;\;l=1,2,\ldots ,M_{x}-1.$$

where

(12) $$r_{i,j}^{x}\left(l\right)=\left\{\begin{array}{c} \frac{-\Delta t}{2c\varrho {\left(\Delta x\right)}^{\alpha +1}}\lambda_{i,l}\omega_{i-j+1}^{\alpha +1}+\frac{-\Delta t}{2c\varrho {\left(\Delta x\right)}^{\alpha }}\lambda_{i,l}^{{}_{x}^{\prime }}\omega_{i-j+l}^{\alpha },\;\;\;j<i-1,\\ \frac{-\Delta t}{2c\varrho {\left(\Delta x\right)}^{\alpha +1}}\left(\lambda_{i,l}\omega_{2}^{\alpha +1}+\lambda_{i,l}\omega_{0}^{\alpha +1}\right)+\frac{-\Delta t}{2c\varrho {\left(\Delta x\right)}^{\alpha }}\left(\lambda_{i,l}^{{}_{x}^{\prime }}\omega_{2}^{\alpha }-\lambda_{i,l}^{{}_{x}^{\prime }}\omega_{0}^{\alpha }\right),\;\;\;j=i-1,\\ \frac{-\Delta t}{2c\varrho {\left(\Delta x\right)}^{\alpha +1}}\left(\lambda_{i,l}\omega_{1}^{\alpha +1}+\lambda_{i,l}\omega_{1}^{\alpha +1}\right)+\frac{-\Delta t}{2c\varrho {\left(\Delta x\right)}^{\alpha }}\left(\lambda_{i,l}^{{}_{x}^{\prime }}\omega_{1}^{\alpha }-\lambda_{i,l}^{{}_{x}^{\prime }}\omega_{1}^{\alpha }\right),\;\;\;j=i,\\ \frac{-\Delta t}{2c\varrho {\left(\Delta x\right)}^{\alpha +1}}\left(\lambda_{i,l}\omega_{0}^{\alpha +1}+\lambda_{i,l}\omega_{2}^{\alpha +1}\right)+\frac{-\Delta t}{2c\varrho {\left(\Delta x\right)}^{\alpha }}\left(\lambda_{i,l}^{{}_{x}^{\prime }}\omega_{0}^{\alpha }-\lambda_{i,l}^{{}_{x}^{\prime }}\omega_{2}^{\alpha }\right),\;\;\;j=i+1,\\ \frac{-\Delta t}{2c\varrho {\left(\Delta x\right)}^{\alpha +1}}\lambda_{i,l}\omega_{j-i+1}^{\alpha +1}-\frac{-\Delta t}{2c\varrho {\left(\Delta x\right)}^{\alpha }}\lambda_{i,l}^{{}_{x}^{\prime }}\omega_{j-i+l}^{\alpha },\;\;\;j>i+1. \end{array}\right.$$

(13) $$r_{i,j}^{y}\left(l\right)=\left\{\begin{array}{c} \frac{-\Delta t}{2c\varrho {\left(\Delta y\right)}^{\beta +1}}\lambda_{l,i}\omega_{i-j+1}^{\beta +1}+\frac{-\Delta t}{2c\varrho {\left(\Delta y\right)}^{\alpha }}\lambda_{i,l}^{{}_{y}^{\prime }}\omega_{i-j+l}^{\beta },\;\;\;j<i-1,\\ \frac{-\Delta t}{2c\varrho {\left(\Delta y\right)}^{\beta +1}}\left(\lambda_{l,i}\omega_{2}^{\beta +1}+\lambda_{l,i}\omega_{0}^{\beta +1}\right)+\frac{-\Delta t}{2c\varrho {\left(\Delta y\right)}^{\beta }}\left(\lambda_{l,i}^{{}_{y}^{\prime }}\omega_{2}^{\beta }-\lambda_{l,i}^{{}_{y}^{\prime }}\omega_{0}^{\beta }\right),\;\;\;j=i-1,\\ \frac{-\Delta t}{2c\varrho {\left(\Delta y\right)}^{\beta +1}}\left(\lambda_{l,i}\omega_{1}^{\beta +1}+\lambda_{l,i}\omega_{1}^{\beta +1}\right)+\frac{-\Delta t}{2c\varrho {\left(\Delta y\right)}^{\beta }}\left(\lambda_{l,i}^{{}_{y}^{\prime }}\omega_{1}^{\beta }-\lambda_{l,i}^{{}_{y}^{\prime }}\omega_{1}^{\beta }\right),\;\;\;j=i,\\ \frac{-\Delta t}{2c\varrho {\left(\Delta y\right)}^{\beta +1}}\left(\lambda_{l,i}\omega_{0}^{\beta +1}+\lambda_{l,i}\omega_{2}^{\beta +1}\right)+\frac{-\Delta t}{2c\varrho {\left(\Delta y\right)}^{\beta }}\left(\lambda_{l,i}^{{}_{y}^{\prime }}\omega_{0}^{\beta }-\lambda_{l,i}^{{}_{y}^{\prime }}\omega_{2}^{\beta }\right),\;\;\;j=i+1,\\ \frac{-\Delta t}{2c\varrho {\left(\Delta y\right)}^{\beta +1}}\lambda_{l,i}\omega_{j-i+1}^{\beta +1}-\frac{-\Delta t}{2c\varrho {\left(\Delta y\right)}^{\beta }}\lambda_{i,l}^{{}_{y}^{\prime }}\omega_{j-i+l}^{\beta },\;\;\;j>i+1. \end{array}\right.$$

Now we define two block matrices, *S* and *H*. First, we create the matrix *S* of dimension $\left[\left(M_{y}-1\right)\cdot \left(M_{x}-1\right)\right]\times \left[\left(M_{y}-1\right)\cdot \left(M_{x}-1\right)\right]$, which is a diagonal block matrix containing matrices $R_{x}\left(l\right),\;l=1,2,\ldots ,M_{y}-1$ on the main diagonal, and zeros in other places.

(14) $$\left[\begin{array}{cccc} R_{x}\left(1\right) & 0 & \ldots & 0\\ 0 & R_{x}\left(2\right) & \ldots & 0\\ \vdots & \vdots & \ddots & \vdots \\ 0 & 0 & \ldots & R_{x}\left(M_{y}-1\right) \end{array}\right]$$

Second, we create matrix *H*, which has the same dimension as matrix *S*, in the following form:

(15) $$\left[\begin{array}{ccccccc} r_{1,1}^{y}\left(1\right) & \ldots & 0 & \; & r_{1,M_{y}-1}^{y}\left(1\right) & \ldots & 0\\ \vdots & \ddots & \vdots & \ldots & \vdots & \ddots & \vdots \\ 0 & \ldots & r_{1,1}^{y}\left(M_{x}-1\right) & \; & 0 & \ldots & r_{1,M_{y}-1}^{y}\left(M_{x}-1\right)\\ \; & \vdots & \; & \ddots & \; & \ldots & \\ r_{M_{y}-1,1}^{y}\left(1\right) & \ldots & 0 & \; & r_{M_{y}-1,M_{y}-1}^{y}\left(1\right) & \ldots & 0\\ \vdots & \ddots & \vdots & \ldots & \vdots & \ddots & \vdots \\ 0 & \ldots & r_{M_{y}-1,1}^{y}\left(M_{x}-1\right) & \; & 0 & \ldots & r_{M_{y}-1,M_{y}-1}^{y}\left(M_{x}-1\right) \end{array}\right]$$

Now it is possible to write the difference scheme (11) in matrix form:

(16) $$\left(I+S+H\right)u^{k+1}=\left(I-S-H\right)u^{k}+\frac{\Delta t}{c\varrho }f^{k+\frac{1}{2}},\;\;\;k=0,1,\ldots$$

where

$$u^{k}={\left[u_{1,1}^{k},u_{2,1}^{k},\ldots ,u_{M_{x}-1,1}^{k},\ldots ,u_{1,M_{y}-1}^{k},u_{2,M_{y}-1}^{k},\ldots u_{M_{x}-1,M_{y}-1}\right]}^{T},$$

$$f^{k+\frac{1}{2}}={\left[f_{1,1}^{k+\frac{1}{2}},f_{2,1}^{k+\frac{1}{2}},\ldots ,f_{M_{x}-1,1}^{k+\frac{1}{2}},\ldots ,f_{1,M_{y}-1}^{k+\frac{1}{2}},f_{2,M_{y}-1}^{k+\frac{1}{2}},\ldots ,f_{M_{x}-1,M_{y}-1}^{k+\frac{1}{2}}\right]}^{T}.$$

The matrices from the difference scheme (16) are large, so the obtainedsystem of equations is time-consuming to solve. Hence, we applied the alternating direction implicit method (ADIM) to the difference scheme (11), which significantly reduces the computation time (details can be found in [27]). This is an important issue in the case of inverse problems, where a direct problem should be solved many times. Let us write the scheme (11) in the form of the directional separation product:

(17) $$\begin{array}{c} \left(1-\frac{\Delta }{c\varrho }{\bar{\delta }}_{x}^{\alpha +1}-\frac{\Delta }{c\varrho }\delta_{x}^{\alpha }\right)\left(1-\frac{\Delta }{c\varrho }{\bar{\delta }}_{y}^{\beta +1}-\frac{\Delta }{c\varrho }\delta_{y}^{\beta }\right)u_{i,j}^{k+1}\\ =\left(1+\frac{\Delta }{c\varrho }{\bar{\delta }}_{x}^{\alpha +1}+\frac{\Delta }{c\varrho }\delta_{x}^{\alpha }\right)\left(1+\frac{\Delta }{c\varrho }{\bar{\delta }}_{y}^{\beta +1}+\frac{\Delta }{c\varrho }\delta_{y}^{\beta }\right)u_{i,j}^{k}+\frac{\Delta t}{c\varrho }f_{i,j}^{k+\frac{1}{2}},\\ i=1,2,\ldots ,M_{x}-1,\;\;j=1,2,\ldots ,M_{y}-1,\;\;k=0,1,\ldots . \end{array}$$

Numerical scheme (17) is split into two parts and solved, respectively, first in the direction *x*, and afterwards in the direction *y*. With this approach, the resulting matrices for the systems of equations have significantly lower dimensions than in the case of the scheme (11). The numerical algorithm has two main steps:

- For each fixed $y_{j}$, solve the numerical scheme in the direction *x*. As a consequence, we will obtain a temporary solution: ${\tilde{u}}_{i,j}^{k+1}$:
  (18) $$\left(1-\frac{\Delta }{c\varrho }{\bar{\delta }}_{x}^{\alpha +1}-\frac{\Delta }{c\varrho }\delta_{x}^{\alpha }\right){\tilde{u}}_{i,j}^{k+1}=\left(1+\frac{\Delta }{c\varrho }{\bar{\delta }}_{x}^{\alpha +1}+\frac{\Delta }{c\varrho }\delta_{x}^{\alpha }\right)\left(1+\frac{\Delta }{c\varrho }{\bar{\delta }}_{y}^{\beta +1}+\frac{\Delta }{c\varrho }\delta_{y}^{\beta }\right)u_{i,j}^{k}+\frac{\Delta t}{c\varrho }f_{i,j}^{k+\frac{1}{2}},$$
- Then, for each fixed $x_{i}$, solve the numerical scheme in the direction *y*:
  (19) $$\left(1-\frac{\Delta }{c\varrho }{\bar{\delta }}_{y}^{\beta +1}-\frac{\Delta }{c\varrho }\delta_{y}^{\beta }\right)u_{i,j}^{k+1}={\tilde{u}}_{i,j}^{k+1}.$$

**Figure 1:**
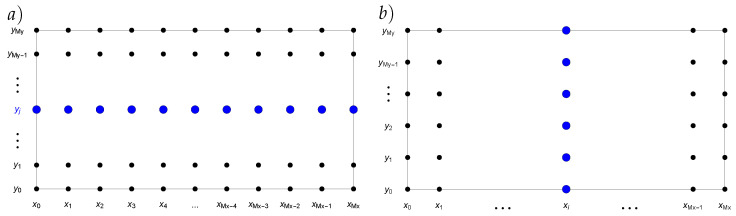
Numerical solution in horizontal direction (for a fixed node $y_{j}$) (**a**) and vertical direction (for a fixed node $x_{i}$) (**b**).

This process can be symbolically depicted as in Figure 1. For the boundary nodes and the initial condition, we applied:

$$u_{0,j}^{k+1}=u_{M_{x},j}^{k+1}=u_{i,0}^{k+1}=u_{i,M_{y}}^{k+1}=0,$$

$$u_{i,j}^{0}=\varphi \left(i\Delta x,j\Delta y\right)=\varphi_{i,j}.$$

In the case of the ADIM method, it is also possible to present the equations in a matrix form, which has been executed below. First, for each $l=1,2,\ldots ,M_{x}-1$, we define auxiliary vectors $u_{l}^{*}$:

(20) $$\left(I-R_{y}\left(l\right)\right)u_{l}^{k}=u_{l}^{*},$$

where $u_{l}^{k}={\left[u_{l,1}^{k},u_{l,2}^{k},\ldots ,u_{l,M_{y}-1}^{k}\right]}^{T},\;\;u_{l}^{*}={\left[u_{l,1}^{*k},u_{l,2}^{*k},\ldots u_{l,M_{y}-1}^{*k}\right]}^{T}$. Hence, we obtain an auxiliary matrix $U^{*k}=\left(u_{i,j}^{*k}\right)$ dimension $\left(M_{x}-1\right)\times \left(M_{y}-1\right)$. Then, the numerical scheme (18) can be written in the following matrix form (for $p=1,2,\ldots ,M_{y}-1$):

(21) $$\left(I+R_{x}\left(p\right)\right){\tilde{u}}_{p}^{k}=\left(I-R_{x}\left(p\right)\right)u_{p}^{**}+\frac{\Delta t}{c\varrho }f_{p}^{k+1},$$

where the temporary solution has the form ${\tilde{u}}_{p}^{k}={\left[{\tilde{u}}_{1,p}^{k},{\tilde{u}}_{2,p}^{k},\ldots ,{\tilde{u}}_{M_{x}-1,p}^{k}\right]}^{T}$, and $u_{p}^{**}={\left[u_{1,p}^{*k},u_{2,p}^{*k},\ldots ,u_{M_{x}-1,p}^{*k}\right]}^{T}$, $f_{p}^{k+\frac{1}{2}}={\left[f_{1,p}^{k+\frac{1}{2}},f_{2,p}^{k+\frac{1}{2}},\ldots f_{M_{x}-1,p}^{k+\frac{1}{2}}\right]}^{T}$. We obtain $M_{y}-1$ systems of equations, each of $\left(M_{x}-1\right)\times \left(M_{x}-1\right)$ dimension. Next, we present the scheme (19) in the direction *y* in matrix form (for $l=1,2,\ldots ,M_{x}-1$):

(22) $$\left(I+R_{y}\left(l\right)\right)u_{l}^{k+1}=\left(I-R_{y}\left(l\right)\right){\tilde{u}}_{l}^{*k},$$

where $u_{l}^{k+1}={\left[u_{l,1}^{k+1},u_{l,2}^{k+1},\ldots ,u_{l,M_{y}-1}^{k+1}\right]}^{T}$ and ${\tilde{u}}_{l}^{*k}={\left[{\tilde{u}}_{l,1}^{k},{\tilde{u}}_{l,2}^{k},\ldots ,{\tilde{u}}_{l,M_{y}-1}^{k}\right]}^{T}$. At this stage of the algorithm, we can solve $M_{x}-1$ systems of equations with dimensions $\left(M_{y}-1\right)\times \left(M_{y}-1\right)$ each. The Bi-CGSTAB [28,29] method is used to solve the equation systems, which has significance influences on the computation time. More implementation details and a comparison of times for the described method can be found in the papers [27,30].

## 3. Inverse Problem

In many engineering problems, in particular in various types of simulations and mathematical modeling, there is a need to solve the inverse problem. In this case, the inverse problem consists of selecting the appropriate model input parameters (1) and (2) to obtain the desired data on the output. Values of the state function *u* at selected points (so-called measurement points) of the domain are treated as input data for the inverse problem. The task consists of selecting unknown parameters of the model in such a way that the *u* function assumes the given values at the measurement points. Problems of this type are badly conditioned, which may result in the instability of the solution or the ambiguity of it [31,32]. Details of the solving algorithm are presented in the following sections.

### 3.1. Parameter Identification

**Figure 2:**
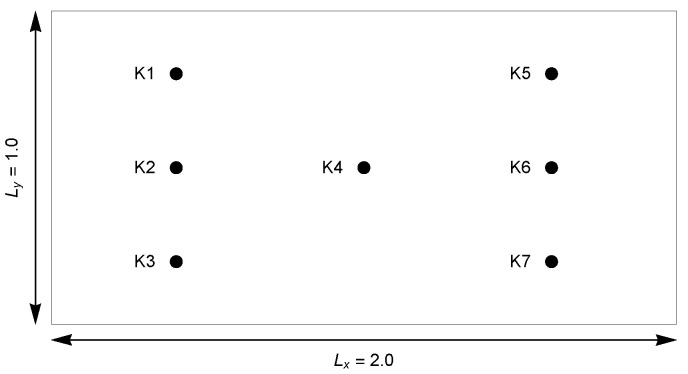
Arrangements of measuring points.

In the model (1) and (2), the following data are assumed:

(23) ϱ=2100,c=900,β=0.6,φ(x,y)=u(x,y,0)=0,

(24) $$\begin{array}{c} f\left(x,y,t\right)=\frac{3,000,000}{1309}\left(82,467{\left(x-2\right)}^{2}x^{2}{\left(y-1\right)}^{2}y^{3}\cos\left(\frac{t}{100}\right)\right.\\ \left.-\frac{1904\sqrt[{5}]{x}\left(25x^{2}-55x+22\right){\left(y-1\right)}^{2}y^{3}\sin\left(\frac{t}{100}\right)}{\Gamma \left(\frac{1}{5}\right)}\right.\\ \left.-\frac{1904\sqrt[{5}]{2-x}\left(25x^{2}-45x+12\right){\left(y-1\right)}^{2}y^{3}\sin\left(\frac{t}{100}\right)}{\Gamma \left(\frac{1}{5}\right)}\right.\\ \left.-\frac{220{\left(x-2\right)}^{2}x^{2}\left(125y^{2}-170y+51\right)y^{7/5}\sin\left(\frac{t}{100}\right)}{\Gamma \left(\frac{2}{5}\right)}\right.\\ \left.-\frac{44{\left(x-2\right)}^{2}x^{2}{\left(1-y\right)}^{2/5}\left(625y^{3}-600y^{2}+90y+4\right)\sin\left(\frac{t}{100}\right)}{\Gamma \left(\frac{2}{5}\right)}\right), \end{array}$$

where (x,y,t)∈[0,2]×[0,1]×[0,200]. The inverse problem deals with finding the λ and α parameters appropriately. The input data for the inverse problem are values of the *u* function at selected points in the area. Additionally, in order to test the algorithm, the following is assumed:

- Location of the measuring points (see Figure 2):
  {K1(0.4,0.8),K2(0.4,0.5),K3(0.4,0.2),K4(1.0,0.5),
  K5(1.6,0.8),K6(1.6,0.5),K7(1.6,0.2)}.
- Two different grids ($M_{x}\times M_{y}\times N$):
  – 160×160×250 (Δx=0.0125,Δy=0.00625,Δt=0.8),
  100×100×200 (Δx=0.02,Δy=0.01,Δt=1.0),
- Different levels of measurement data disturbances (errors with a normal distribution): 0%, 2%, 5%, 10%.

To solve the problem, we create an objective function that compares the values of the *u* function calculated for the determined values of the searched parameters λ,α (at measurement points) with the measurement data. Therefore, we define the objective function as follows:

(25) $$J\left(\lambda ,\alpha \right)=\sum_{i,j}^{N_{1}}\sum_{k}^{N_{2}}{\left(u_{i,j}^{k}\left(\lambda ,\alpha \right)-{\overset{m}{u}}_{i,j}^{k}\right)}^{2},$$

where $N_{1}$ and $N_{2}$ are the number of measuring points and the number of measurements in a given measuring point, respectively. In the considered example, $N_{1}=7$, and $N_{2}$ depends on the used mesh. By $u_{i,j}^{k}\left(\lambda ,\alpha \right)$, we denote the values of the *u* function obtained in the algorithm for the fixed parameters λ,α, and by ${\overset{m}{u}}_{i,j}^{k}$ measurement data. Finding the minimum of the objective function (25), we find the sought parameters.

### 3.2. Function Minimization

In the case of the minimization objective function, we can use any heuristic algorithm (e.g., swarming algorithms). In this paper, we decided to use two algorithms:

- Ant colony optimization algorithm (ACO).
- Hooke–Jeeves algorithm (HJ).

In this section, we describe both algorithms.

#### 3.2.1. Ant Colony Optimization Algorithm

The presented ACO algorithm is a probabilistic one, so we obtain a different result in each execution. Proper selection of algorithm parameters should make the obtained results give convergent solutions. The algorithm is inspired by the behavior of an ant swarm in nature. More about the ACO algorithm and its applications can be found in the articles [33,34,35]. In order to describe the algorithm, we introduce the following notations:

J—objective function,n—domain size,

nT—number of threads,M=nT·p—number of ants in the population,

I—number of iterations,L—number of pheromone spots,

q,ξ—algorithm parameters selected empirically.

Algorithm 1 presents ACO algorithm step by step. Number of execution objective function in case of ACO algorithm is equal to L+M·I.

#### 3.2.2. Hooke–Jeeves Algorithm

The Hooke–Jeeves algorithm is a deterministic algorithm for searching for the minimum of an objective function. It is based on two main operations:

- Exploratory move. It is used to test the behavior of the objective function in a small selected area with the use of test steps along all directions of the orthogonal base.
- Pattern move. It consists of moving in a strictly determined manner to the next area where the next trial step is considered, but only if at least one of the steps performed was successful.

In this algorithm, we consider the following parameters:

$$[d^{1},d^{2},\ldots ,d^{n}]—\mathrm{orthogonal}\;\mathrm{basis}\;\mathrm{of}\;\mathrm{vectors}\;\mathrm{in}\;\mathrm{the}\;\mathrm{considered}\;\mathrm{space},$$

τ—steps length vector,ξ—accuracy of calculations (stop condition),

β∈[0,1]—parameter narrowing the steps τ,

$$x^{0}=\left[x_{1},x_{2},\ldots ,x_{n}\right]—\mathrm{starting}\;\mathrm{point}$$

Pseudocode for the Hooke–Jeeves method is presented in Algorithm 2. The only drawback of the discussed method is the possibility of falling into the local minimum with more complicated objective functions. More details about the algorithm itself and its applications can be found in the papers [36,37].
**Algorithm 1** Ant Colony Optimization algorithm (ACO).1:Initialization part.2:Random generation of *L* vectors from the domain of solving problem (the so-called pheromone spots): $x^{i}=\left[x_{1}^{i},x_{2}^{i},\ldots ,x_{n}^{i}\right]$  (i=1,2,…,L).3:Calculating the value of the objective function for each of the pheromone spot (for each solution vector).4:Sorting the set of solutions in descending order by the quality of solutions (the lower the value of the objective function, the better the solution). Each solution is assigned an index.5:Iterative part.6:**for** *iteration* = 1, 2, …, *I* **do**7:    Each pheromone spot (solution vector) is assigned a probability according to the formula:
$$p_{l}=\frac{\omega_{l}}{\sum_{l=1}^{L}\omega_{l}}\;\;\;l=1,2,\ldots ,L,$$
where $\omega_{l}$ are weights related to the solution index *l* and expressed by the formula:
$$\omega_{l}=\frac{1}{qL\sqrt{2\pi }}\cdot e^{\frac{-{\left(l-1\right)}^{2}}{2q^{2}L^{2}}}.$$8:    **for** *k* = 1, 2, …, *M* **do**9:          Ant randomly chooses the *l*-th solution with a probability of $p_{l}$.10:        Then ant transforms each of the coordinates (j=1,2,…,n) of the selected solution using Gauss function:
$$g\left(x,\mu ,\sigma \right)=\frac{1}{\sigma \sqrt{2\pi }}\cdot e^{\frac{-{\left(x-\mu \right)}^{2}}{2\sigma^{2}}},$$
where $\mu =s_{j}^{l},\;\sigma =\frac{\xi }{L-1}\sum_{p=1}^{L}\left|s_{j}^{p}-s_{j}^{l}\right|$.11:    **end for**12:    *M* new solutions are obtained. Divide set of new solutions into nT groups and calculate value of objective function *J* for each solution in each group in separate thread.13:    From the two sets of solutions (new one and previous one) remove *M* worst solutions and rest sort according to the quality (value of objective function).14:**end for**

**Algorithm 2** Hooke–Jeeves algorithm (pseudocode).
1:Search the space around the current point $x^{k}$ along directions from the orthogonal base $\left[d^{1},d^{2},\ldots ,d^{n}\right]$ with step $\tau^{i}$ (i=1,2,…,n). This is an exploratory move.2:If a better point is found, continue in that direction. This is a pattern move.3:If no better point is found then narrow down the search space using the narrowing parameter β.


## 4. Results—Numerical Examples

We consider the inverse problem described in the Section 3.1. In the models (1) and (2), we set data described by the Equations (23) and (24). We used two different grids 160×160×250 and 100×100×200 and different levels of measurement data disturbances (input data for the inverse problem): 0%, 2%, 5%, 10%. The unknown data in the model are λ and α—these data need to be identified using the presented algorithm. To examine and test the algorithm, we know exact values of these parameters, which are λ=240, α=0.8.

First, we present the results obtained using the ACO algorithm. We set the following parameters of the ant algorithm:

λ∈[100,500],α∈(0.01,0.99),

L=16,M=32,I=20,nT=4.

Based on the L,M,I parameters, we can determine the number of calls to the objective function, which in our example is M·I+L=656. Obtained results are presented in Table 1. The best results were obtained for exact input data and 100×100×200 mesh, the relative errors of reconstruction parameters λ and α are 0.0283% and 0.584%, respectively, and for the 160×160×250, mesh these errors are equal to 0.151% and 0.687%. In the case of the input data with a pseudo-random error, the obtained results are also very good, and the errors of reconstructed parameters do not exceed the input data disturbance errors. In particular, the errors of reconstruction of the λ coefficient are very small and do not exceed 1% (except in the case of disturbing the input data with an error of 10% and the 100×100×200 grid). Relative errors of reconstructed α parameter have values greater than λ errors, most likely due to the fact that the sought value is significantly lower than λ. Of course, along with the increase in input data disturbances, the values of the minimized objective function also increased. Except for in a few cases, the mesh density did not significantly affect the results.

**Figure 3:**
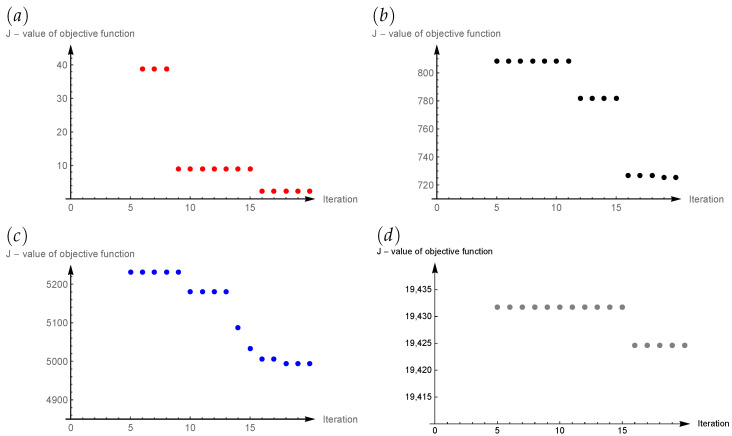
Values of objective function *J* in iterations of ACO algorithm for different levels of input data noise: (**a**) 0%, (**b**) 2%, (**c**) 5%, (**d**) 10%.

Figure 3 shows how the value of the objective function changed depending on the iteration number for four input data cases. The figures do not include the objective function values for the initial iterations. This is due to the fact that these values were relatively high, and inclusion in the figures would reduce their legibility. We can see that in the last few iterations (2–5), the values of the objective function do not change anymore. The appropriate selection of the L, M, I parameters for the ACO algorithm affects the computation time and is not always a simple task. It depends on the complication of the objective function and the number of sought parameters (size of the problem). In particular, a situation in which the algorithm does not change the solution in the next dozen iterations should be avoided. As we can observe in the presented example, the selection of ACO parameters, such as the number of iterations, as well as the size of the population, seems appropriate.

For comparison, we now use the deterministic Hooke–Jeeves algorithm. The following parameters are set in it:

orthogonal basis of vectors:{[1,0],[0,1]}

$$\mathrm{vector}\;\mathrm{of}\;\mathrm{steps}:\tau =\left[\tau_{\lambda },\tau_{\alpha }\right]=\left[4,0.05\right]$$

narrowing parameter:β=0.5,stop criterion:ξ=0.0001.

It is a deterministic algorithm, and the resulting solution, as well as the number of calls to the objective function, depend on the starting point and stop criterion ξ. In our example, we consider four different starting points: (100,0.2), (300,0.1), (450,0.5), (500,0.9). It turned out that regardless of the selected starting point, the same solution was always obtained, but it should be noted that in the case that the value of any of the reconstructed parameters exceeded the predetermined limits, then we execute the so-called penalty function. It was significant in the case of the (100,0.2) starting point, for which the algorithm exceeded the limits and stopped at the local minimum; e.g., for the 160×160×250 grid and 0% disturbances, we obtained the results $\bar{\lambda }\approx 250,\;\bar{\alpha }\approx 1.8,\;J\approx 138$. Similar results were obtained for the remaining cases and the (100,0.2) start. Table 2 shows the results obtained using the Hooke–Jeeves algorithm. Comparing the results obtained from both algorithms, we can see that in most cases the errors in reconstruction of the parameters are smaller for the Hooke–Jeeves algorithm; e.g., for the 160×160×250 and 2% input data disturbance errors, errors in sought parameters λ and α for the HJ algorithm were 0.0198% and 0.231%, respectively, while for the ACO algorithm, these errors were 0.371% and 1.64%. In addition, the value of the objective function for the HJ algorithm was smaller $J_{HJ}\approx 1014$, $J_{ACO}\approx 1020$. As mentioned earlier, the failure to apply the penalty function caused the HJ algorithm for the (100,0.2) starting point to return unsatisfactory results. This should be noted when the objective function is complicated, for example, by increasing the number of parameters to be found.

Now we present the error of reconstruction of the *u* state function in the grid points. These results are summarized in Table 3. The mean errors of reconstruction of the *u* state function are at a low level and do not exceed 0.5% in each of the analyzed cases. We can also observe that the maximum errors in most cases are greater for the 100×100×200 grid; in particular, it is visible for the input data noised by the 5% and 10% errors.

**Figure 4:**
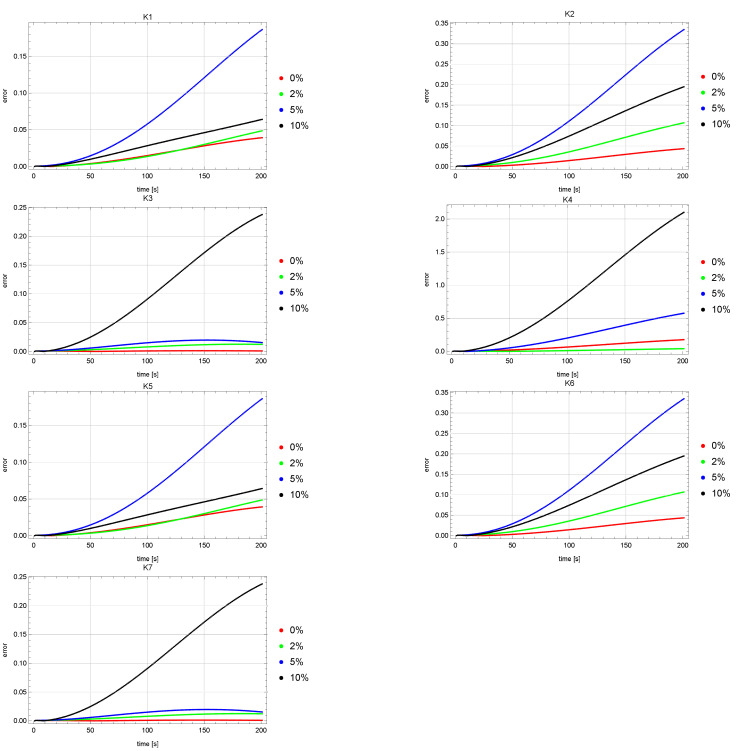
Errors of reconstruction of *u* state function in points K1, K2, K3, K4, K5, K6, K7 for ACO algorithm.

**Figure 5:**
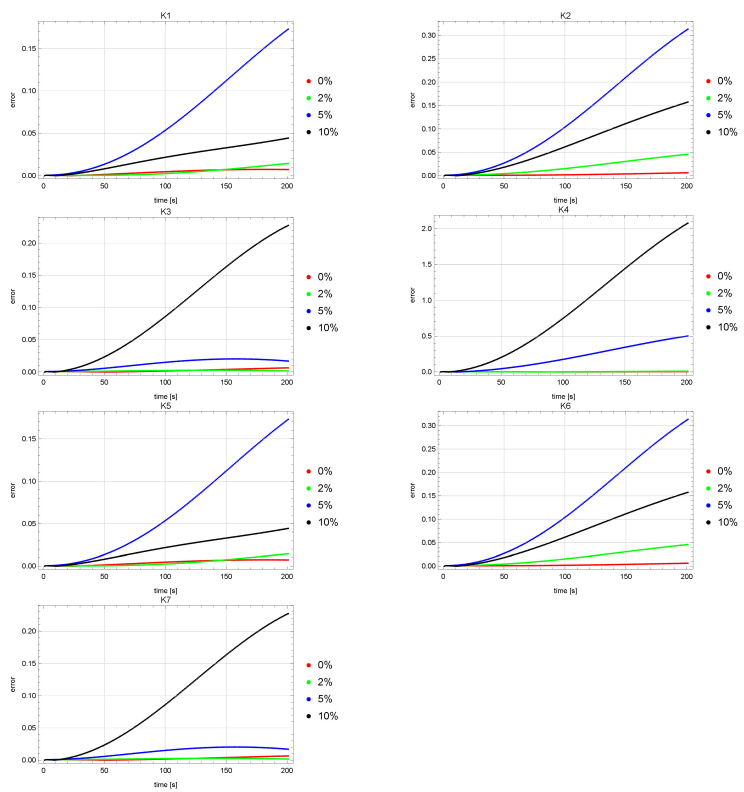
Errors of reconstruction of *u* state function in points K1, K2, K3, K4, K5, K6, K7 for HJ algorithm.

Figure 4 and Figure 5 show error plots of reconstruction of the *u* state function at the measurement points K1, K2, …, K7. The graphs of these errors for both the ACO and HJ algorithms are quite similar. It can be noticed that for the measurement points K1, K2, K5, K6, greater errors were obtained for the input data noised by the 5% error than for the input data disturbed by the error of 10%. Levels of the *u* reconstruction errors for the input data unaffected and affected by the 2% error (red and green colors) are on a much lower level than for the other input data (blue and black colors).

### Sensitivity Analysis

A sensitivity analysis was also performed for both reproduced parameters [38]. Sensitivity coefficients are derived from the measured quantity according to the reproduced quantity:

(26) $$\begin{array}{cc} Z_{\alpha } & =\frac{\partial u(x,y,t)}{\partial \alpha }, \end{array}$$

(27) $$\begin{array}{cc} Z_{\lambda } & =\frac{\partial u(x,y,t)}{\partial \lambda }. \end{array}$$

In the calculations, both of the above derivatives are approximated by central difference quotients:

(28) $$\begin{array}{cc} Z_{\alpha } & \approx \frac{u_{\alpha +\varepsilon }\left(x,y,t\right)-u_{\alpha -\varepsilon }\left(x,y,t\right)}{2\;\varepsilon }, \end{array}$$

(29) $$\begin{array}{cc} Z_{\lambda } & \approx \frac{u_{\lambda +\varepsilon }\left(x,y,t\right)-u_{\lambda -\varepsilon }\left(x,y,t\right)}{2\;\varepsilon }, \end{array}$$

where $\varepsilon =10^{-5}$ [39], and $u_{p}\left(x,y,t\right)$ denotes the state function determined for a given value of *p*.

**Figure 6:**
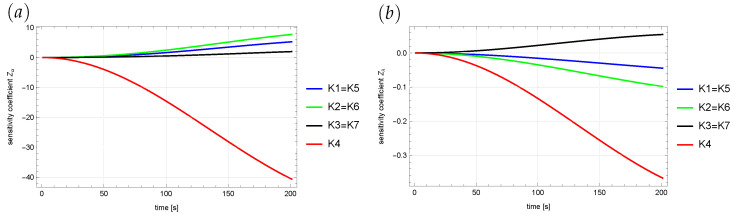
Sensitivity coefficient in measurement points along the time domain: (**a**) $Z_{\alpha }$, (**b**) $Z_{\lambda }$.

We considered a test case with α=0.8 and λ=240. Figure 6 shows the variability of the sensitivity coefficients at measurement points over the entire analyzed period of time. The obtained results were symmetrical with respect to the vertical axis of symmetry of the area—the line x=1. Therefore, the measurement coefficients in points $K_{5}$, $K_{6}$, and $K_{7}$ are equal to the coefficients in points $K_{1}$, $K_{2}$, and $K_{3}$, respectively. The performed sensitivity analysis showed that the positions selected for the measurement points are correct. They ensure the appropriate sensitivity of the state function to changes in the values of the restored parameters.

## 5. Conclusions

This paper presents algorithms for direct and inverse solutions for a model consisting of a differential equation with a fractional derivative with respect to a space of the Riemann–Liouville type. Equations of this type are used to describe the phenomena of anomalous diffusion, e.g., anomalous heat transfer in porous media. The inverse problem has been reduced to the search for the minimum of a properly created objective function. Two algorithms were used to deal with this problem: ant colony optimization algorithm and Hooke–Jeeves method. From the presented numerical example, we can draw the following conclusions:

- The obtained results are satisfactory and errors of parameters reconstruction are minimal.
- Both presented algorithms returned similar results, but in the case of the HJ algorithm, it was necessary to use the penalty function for one of the starting points.
- The number of evaluation of the objective function was smaller for the HJ algorithm (250–300) than for the ACO algorithm (656).

The used differential scheme is unconditionally stable and has the approximation order equal to $O({\left(\Delta x\right)}^{2}+{\left(\Delta y\right)}^{2}+{\left(\Delta t\right)}^{2})$ [26]. The convergence of the differential scheme is fast; already for sparse meshes, the approximation errors for the solution of the direct problem are small [27]. In addition, in the case of the inverse problem considered in this paper, it is enough to use a relatively sparse mesh to very well reconstruct the searched parameters. The presented method can be used as a tool for parameter training in artificial neural networks.

## Figures and Tables

**Table 1:** Results of calculations in case of ACO algorithm. $\bar{\lambda }$—reconstructed value of thermal conductivity coefficient; $\bar{\alpha }$—reconstructed value of *x*-direction derivative order; δ—the relative error of reconstruction; *J*—the value of objective function; σ—standard deviation of objective function.

Mesh Size	Noise	$\bar{\lambda }$	$\delta_{\bar{\lambda }}\left[\%\right]$	$\bar{\alpha }$	$\delta_{\bar{\alpha }}\left[\%\right]$	*J*	$\sigma_{J}$
100 × 100 × 200	0%	240.06	2.83 × 10^−2^	0.8046	5.84 × 10^−1^	2.24	8.72
	2%	240.71	2.95 × 10^−1^	0.7934	8.14 × 10^−1^	725.13	5.23
	5%	241.49	6.21 × 10^−1^	0.7735	3.31	4994.21	14.72
	10%	236.61	1.41	0.7798	2.52	19,424.61	6.44
160 × 160 × 250	0%	239.63	1.51 × 10^−1^	0.8054	6.87 × 10^−1^	1.72	19.17
	2%	239.11	3.71 × 10^−1^	0.8131	1.64	1020.84	11.39
	5%	241.28	5.36 × 10^−1^	0.7943	7.03 × 10^−1^	5396.34	5.41
	10%	241.76	7.34 × 10^−1^	0.7761	2.98	23,675.2	2.66

**Table 2:** Results of calculations in case of Hooke–Jeeves algorithm: $\bar{\lambda }$ —reconstructed value of thermal conductivity coefficient; $\bar{\alpha }$—reconstructed value of *x*-direction derivative order; δ—the relative error of reconstruction; *J*—the value of objective function; $f_{e}$—number of evaluation objective function; SP—starting point.

Mesh Size	Noise	SP	$\bar{\lambda }$	$\delta_{\bar{\lambda }}\left[\%\right]$	$\bar{\alpha }$	$\delta_{\bar{\alpha }}\left[\%\right]$	*J*	$f_{e}$
100 × 100 × 200	0%	(100, 0.2)	240.15	6.57 × 10^−2^	0.7993	8.33 × 10^−2^	0.0182	272
		(300, 0.1)						246
		(450, 0.5)						240
		(500, 0.9)						299
	2%	(100, 0.2)	240.38	1.59 × 10^−1^	0.7971	3.61 × 10^−1^	724.57	254
		(300, 0.1)						217
		(450, 0.5)						235
		(500, 0.9)						270
	5%	(100, 0.2)	241.44	6.03 × 10^−1^	0.7757	3.03	4993.85	230
		(300, 0.1)						203
		(450, 0.5)						257
		(500, 0.9)						255
	10%	(100, 0.2)	236.86	1.31	0.7781	2.73	19,424.36	217
		(300, 0.1)						199
		(450, 0.5)						239
		(500, 0.9)						245
160 × 160 × 250	0%	(100, 0.2)	240.06	2.51 × 10^−2^	0.7997	3.21 × 10^−2^	0.0036	265
		(300, 0.1)						225
		(450, 0.5)						221
		(500, 0.9)						292
	2%	(100, 0.2)	239.95	1.98 × 10^−2^	0.8018	2.31 × 10^−1^	1014.21	257
		(300, 0.1)						231
		(450, 0.5)						233
		(500, 0.9)						284
	5%	(100, 0.2)	240.85	3.55 × 10^−1^	0.7935	8.11 × 10^−1^	5393.44	241
		(300, 0.1)						213
		(450, 0.5)						243
		(500, 0.9)						266
	10%	(100, 0.2)	241.44	6.02 × 10^−1^	0.7817	2.28	23,673.38	255
		(300, 0.1)						227
		(450, 0.5)						273
		(500, 0.9)						280

**Table 3:** Errors of reconstruction function *u* in grid points in case of reconstruction of two parameters λ,α ($\Delta_{\mathrm{avg}}$—average absolute error; $\Delta_{\max}$—maximal absolute error).

Algorithm	Errors	Mesh 100 × 100 × 200			
		0%	2%	5%	10%
ACO	Δ_avg_[K]	3.04 × 10^−2^	2.94 × 10^−2^	1.37 × 10^−1^	2.59 × 10^−1^
	Δ_max_[K]	1.95 × 10^−1^	2.68 × 10^−1^	1.13	2.46
HJ	Δ_avg_[K]	6.28 × 10^−3^	1.36 × 10^−2^	1.24 × 10^−1^	2.59 × 10^−1^
	Δ_max_[K]	1.11 × 10^−1^	1.24 × 10^−1^	1.04	2.42
		mesh 160 × 160 × 250			
		0%	2%	5%	10%
ACO	Δ_avg_[K]	2.77 × 10^−2^	6.55 × 10^−2^	4.65 × 10^−2^	1.77 × 10^−1^
	Δ_max_[K]	2.19 × 10^−1^	5.27 × 10^−1^	3.11 × 10^−1^	9.96 × 10^−1^
HJ	Δ_avg_[K]	2.68 × 10^−3^	1.08 × 10^−2^	3.36 × 10^−2^	8.84 × 10^−2^
	Δ_max_[K]	4.72 × 10^−2^	7.43 × 10^−2^	2.53 × 10^−1^	7.55 × 10^−1^

## Nomenclature

The following abbreviations are used in this manuscript:

*c*	specific heat
$d^{i}$	*i*-th vector in orthogonal base in HJ method
*f*	additional source term
$f_{i,j}^{k}$	value of function *f* in point $\left(t_{k},x_{i},y_{j}\right)$
*g*	auxiliary coefficient to determine ω
*I*	number of iterations in ACO algorithm
*J*	objective function
$K_{i}$	*i*-th measurement point
*L*	number of pheromone spots in ACO algorithm
$L_{x}$	length in *x*-direction
$L_{y}$	length in *y*-direction
$M_{x}$	mesh size in *x*-direction
$M_{y}$	mesh size in *y*-direction
*n*	number of sought parameters in ACO algorithm
nT	number of threads in ACO algorithm
*N*	mesh size in time
$r_{i,j}^{x},r_{i,j}^{y}$	coefficients of matrices $R_{x},R_{y}$
$R_{x},R_{y}$	auxiliary matrices to to describe the solution of a direct problem
*t*	time
*u*	state function (temperature)
$u_{i,j}^{k}$	value of state function in point $\left(t_{k},x_{i},y_{j}\right)$
*q*	parameter in ACO algorithm
*x*	spatial variable
$x_{i}$	value of *x* variable for iΔx
$x^{0}$	starting point in HJ method
*y*	spatial variable
$y_{j}$	value of *y* variable for jΔy
*T*	final moment of time
$t_{k}$	value of time for kΔt
Greek Symbols	
α	order of derivative in *x*-direction
β	order of derivative in *y*-direction
Γ	gamma function
$\delta_{x}^{\alpha },{\bar{\delta }}_{x}^{\alpha +1},\delta_{y}^{\beta },{\bar{\delta }}_{y}^{\beta +1}$	auxiliary operators to describe the solution of a direct problem
Δt	time step
Δx	step mesh in *x*-direction
Δy	step mesh in *y*-direction
λ	thermal conductivity
$\lambda_{i,j}$	value of thermal conductivity in point $\left(x_{i},y_{j}\right)$
φ	temperature in t=0
ϱ	mass density
ξ	stop criterion in HJ method
τ	steps length vector in HJ method
ω	weight in shifted Grünwald formula
Ω	domain of differential equation
